# Rapid Enabling of *Gluconobacter oxydans* Resistance to High D-Sorbitol Concentration and High Temperature by Microdroplet-Aided Adaptive Evolution

**DOI:** 10.3389/fbioe.2021.731247

**Published:** 2021-09-03

**Authors:** Li Liu, Weizhu Zeng, Shiqin Yu, Jianghua Li, Jingwen Zhou

**Affiliations:** ^1^ National Engineering Laboratory for Cereal Fermentation Technology, Jiangnan University, Wuxi, China; ^2^ Engineering Research Center of Ministry of Education on Food Synthetic Biotechnology, Jiangnan University, Wuxi, China; ^3^ Science Center for Future Foods, Jiangnan University, Wuxi, China; ^4^ Jiangsu Provisional Research Center for Bioactive Product Processing Technology, Jiangnan University, Wuxi, China; ^5^ Jiangsu Province Engineering Research Center of Food Synthetic Biotechnology, Jiangnan University, Wuxi, China

**Keywords:** adaptive evolution, evolutionary strategies, *Gluconobacter oxydans*, microbial microdroplet culture system, MMC

## Abstract

*Gluconobacter oxydans* is important in the conversion of D-sorbitol into l-sorbose, which is an essential intermediate for industrial-scale production of vitamin C. In a previous study, the strain *G. oxydans* WSH-004 could directly produce 2-keto-l-gulonic acid (2-KLG). However, its D-sorbitol tolerance was poor compared with that of other common industrial *G. oxydans* strains, which grew well in the presence of more than 200 g/L of D-sorbitol. This study aimed to use the microbial microdroplet culture (MMC) system for the adaptive evolution of *G. oxydans* WSH-004 so as to improve its tolerance to high substrate concentration and high temperature. A series of adaptively evolved strains, *G. oxydans* MMC1-MMC10, were obtained within 90 days. The results showed that the best strain MMC10 grew in a 300 g/L of D-sorbitol medium at 40°C. The comparative genomic analysis revealed that genetic changes related to increased tolerance were mainly in protein translation genes. Compared with the traditional adaptive evolution method, the application of microdroplet-aided adaptive evolution could improve the efficiency in terms of reducing time and simplifying the procedure for strain evolution. This research indicated that the microdroplet-aided adaptive evolution was an effective tool for improving the phenotypes with undemonstrated genotypes in a short time.

## Introduction

*Gluconobacter* strains belong to the group of acetic acid bacteria (Prust et al., 2005; Deppenmeier and Ehrenreich, 2009). They have a unique capacity to incompletely oxidize polyol substrates to produce a high titer of oxidation products, such as l-sorbose (vitamin C synthesis) (Azar and Alemzadeh, 2020) and 6-(N-hydroxyethyl)-amino-6-deoxy-α-l-sorbofuranose (a key intermediate for the synthesis of miglitol) (Liu et al., 2020). In the current industrial-scale production of vitamin C *via* the two-step fermentation method, *G. oxydans* is used to convert D-sorbitol into l-sorbose, which is the substrate for the second-step fermentation process (Pappenberger and Hohmann, 2014). However, l-sorbose fermentation is severely inhibited by D-sorbitol when the initial concentrations are more than 200 g/L in industry (Giridhar and Srivastava, 2002). Fed-batch fermentation is usually employed to alleviate substrate inhibition, and subsequently to improve l-sorbose productivity. The titer of the final product (l-sorbose) can be reduced and the fermentation time extended with this fermentation process (Zhou et al., 2019). Besides, the application of fed-batch fermentation can greatly increase the production cost and the risk of contamination. It is necessary to obtain a *G. oxydans* strain that can tolerate higher D-sorbitol concentration so as to release the substrate inhibition.

Improving the tolerance of industrial microorganisms to more harsh conditions, such as higher substrate concentration and higher temperature, is a long pursued goal for a more economic industrial fermentation process (Pereira et al., 2019; Wu et al., 2020). Enhanced tolerance to higher substrate/product concentration can not only further improve the final titer of the target product but also significantly simplify the fermentation process by avoiding complicated fed-batch or *in situ* product removing operations (Heerema et al., 2011; Gaber et al., 2021). Higher fermentation temperature can decrease the use of cooling water, thus decreasing the energy cost, and extend effective production time, especially in warmer areas (Wang et al., 2019). Because these two phenotypes play important roles in improving the economy of the fermentation process, researchers have conducted a number of studies that have revealed many important regulatory mechanisms. For example, altering the sterol composition could improve the thermotolerance of *Saccharomyces cerevisiae* (Caspeta et al., 2014), and strengthening the opposing potassium and proton electrochemical membrane gradients could facilitate the general resistance of *S. cerevisiae* to multiple alcohols (Lam et al., 2014). However, up to now, the general mechanisms of thermotolerance in microorganisms are still unclear. Adaptive evolution continues to be of interest to accelerate the breeding of robust microorganisms with improved high-temperature fermentation performance.

For directly improving the tolerance of microorganisms to adverse environmental stresses, adaptive evolution is an effective method to evolve microorganisms under specific environmental conditions, such as tolerance to high substrate/product concentration, high temperature, and low/high pH (d’oelsnitz and Ellington, 2018; Sandberg et al., 2019). Adaptive evolution has many strategies to obtain desired phenotype strains. For example, the classical laboratory evolution is to culture an organism under selective conditions (temperature and presence of inhibitors) so that mutant clones with beneficial mutations are enriched in the population (Rudolph et al., 2010; Ju et al., 2016; Zhu et al., 2018). However, it has inherent limitations because conventional adaptive evolution methods are typically labor-intensive, extremely time-consuming (from months to years generally), with low throughput; and are poorly parallelized. For example, Jin et al. (2019) improved the conversion rate of all nonglucose sugars to the corresponding sugar acids by several folds after 420 days of continuous culture. Recently, advances in miniaturization and parallelization of microbial cultivation systems have translated into advances in the capabilities of microbioreactors, which are now widely applied in studies of evolution (Jakiela et al., 2013; Wong et al., 2018). Compared with traditional microbial cultivation methods, microcultivation systems have many advantages, such as high throughput, less reagent and labor cost, and superior cultivation properties in mixing and parallelization (Jian et al., 2020; Zeng et al., 2020).

In this study, an efficient and reliable adaptive evolution approach was designed to improve the tolerance of *G. oxydans* WSH-004 to high D-sorbitol concentration and high temperature in a microbial microdroplet culture (MMC) system. Within 90 days of continuous adaptive evolution in MMC, ten adaptive evolutionary strains (MMC1–MMC10) were obtained. The optimum evolved strain MMC10 achieved the productivity of 3.13 g/(Lh) in a 5-L fermentor cultured in a medium containing 300 g/L of initial D-sorbitol, which was similar to that of the original strain *G. oxydans* WSH-004 cultured in a medium containing 80 g/L of initial D-sorbitol. Genome sequencing of evolved strains showed that most mutations were related to the translational process, which was rarely mentioned for improving tolerance to stresses in previous studies. The evolved strains and the genes identified could not only be used for more efficient production of l-sorbose and its derivatives but also provide clues for the rational engineering of microorganisms for enhanced tolerances to stresses.

## Materials and Methods

### Strains, Culture Media, and Reagents

*Gluconobacter oxydans* WSH-004, which used D-sorbitol to produce l-sorbose and 2-KLG, was screened in a previous study (Chen et al., 2019). The D-sorbitol medium (10 g/L of yeast extract and 80 g/L of D-sorbitol) was used for culturing the *G. oxydans* strains. In agar plates, 2% agar was added. Adaptive evolution of a D-sorbitol medium (10 g/L of yeast extract and 300 g/L of D-sorbitol) was used for adaptive evolution. Carrier oil (mineral oil with 12 g/L of Span 80; Sigma–Aldrich, Germany) and MMC chips for MMC were purchased from Wuxi Tmaxtree Biotechnology Co., Ltd. (Wuxi, China).

### Adaptive Evolution of *G. oxydans* to High D-Sorbitol Concentration

**FIGURE 1:**
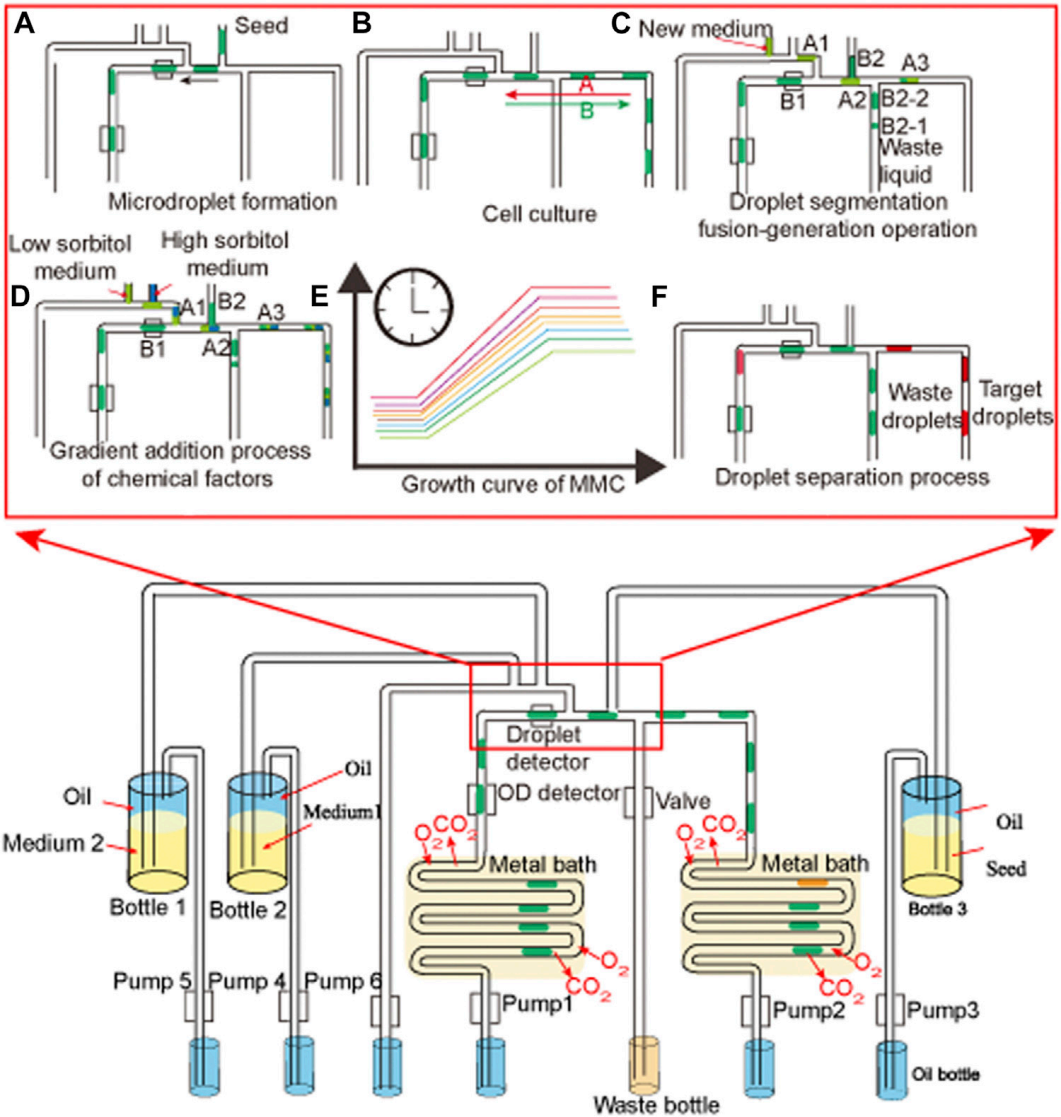
Diagram of the operation of the microdroplet culture system. **(A)** Microdroplet formation, **(B)** cell culture, **(C)** droplet segmentation fusion-generation operation, **(D)** gradient addition process of chemical factors, **(E)** growth curve of MMC, and **(F)** droplet separation process.

*G. oxydans* WSH-004 preserved in glycerol tubes was inoculated to solid D-sorbitol medium plates and cultured for 48 h at 30°C. A single colony was inoculated into 250-mL flasks containing 25 mL of D-sorbitol medium for 48 h at 30°C and 220 rpm. The seed was injected into a special sterilized seed bottle (Figure 1, bottle 3) for adaptive evolution in MMC (Jian et al., 2020). Meanwhile, the D-sorbitol medium was injected into a special sterilized medium bottle (Figure 1, bottle 2), while the adaptive evolution D-sorbitol medium was injected into another special sterilized medium bottle (Figure 1, bottle 1). The first step for adaptive evolution in MMC was the microdroplet formation (Figure 1A), in which pumps 2 and 3 were operated to form 2 μL microdroplets. The second step involved culturing the microdroplets in MMC (Figure 1B), in which pumps 1 and 2 were operated to move the microdroplets in special tubes that could pass through the air but not through water and cells. A key step for the adaptive evolution and continuous culture is the droplet segmentation fusion-generation (Figures 1C,D). After a period of adaptive evolution in MMC, the droplet with a higher OD_600_ value (Figure 1F) was collected and spread on the D-sorbitol medium plates with selective pressure (high D-sorbitol concentration or/and high-temperature conditions).

### Adaptive Evolution of *G. oxydans* to High Temperature

*G. oxydans* MMC4 obtained in the last round of adaptive evolution to the high D-sorbitol concentration was used as the start strain. The single colony of *G. oxydans* MMC4 was inoculated into 250-ml flasks containing 25 ml of D-sorbitol medium for 48 h at 30°C and 220 rpm to get the seeds. All steps were the same (microdroplet formation, incubation, droplet segmentation fusion-generation, and sorting), except for the gradual increase in the metal bath temperature instead of increasing the D-sorbitol concentration.

### Culture Conditions in Shake Flasks

For verifying the tolerance of evolved strains to different D-sorbitol concentrations and temperature, a single colony of the strain was inoculated into 250-ml flasks containing 25 ml of D-sorbitol medium for 48 h at 30°C and 220 rpm for seed culture. Then, 5 ml of the seed culture was transferred to 25 ml of the culture medium containing different D-sorbitol concentrations (50–400 g/L of D-sorbitol, 10 g/L of yeast extract, and 50 μg/ml of cefoxitin). The flasks were then cultured at 220 rpm at 30, 35, or 40°C.

### Genome Sequencing and Data Analysis

A single colony of the evolved strains isolated from the evolved populations was cultivated for 36–48 h in 25 ml of D-sorbitol medium until the OD_600_ value reached 3. A genomic DNA extraction kit (Sangon Biotech, Shanghai) was used to extract genomic DNA of *G. oxydans* strains using the protocol recommended by the manufacturer. High-throughput genome sequencing was performed with the Illumina Hiseq using 2 × 150 paired-end reads targeting a genome. The average coverage for each sample was more than 300X. Genome sequencing was performed by Sangon Biotech. Referring to the GATK best practice–recommended design process, BWA was used to compare the effective data of the sample to the reference genome (Kumar et al., 2019). The SAM tool was used to compare the results for format conversion and sorting, and statistical comparison results (Li et al., 2009). The HaplotypeCaller of GATK was employed to analyze the genetic differences between the adaptive evolution strains and wild-type strain, merge and integrate the analysis of different samples, and obtain sample variation information (McKenna et al., 2010).

### Culture Conditions in Bioreactors

Fermentation studies were performed in a 5-L bioreactor (T&J Bioengineering, Shanghai, China) with a working volume of 3 L. For batch fermentation, a single colony of the strain was inoculated into 500-ml flasks containing 50 ml of D-sorbitol medium for 48 h at 30°C and 220 rpm. Then, 300 ml of the seed culture was inoculated into a bioreactor with 2.7 L of the culture medium containing different D-sorbitol concentrations (300 g/L of D-sorbitol and 10 g/L of yeast extract, or 80 g/L of D-sorbitol and 10 g/L of yeast extract). The temperature was maintained at 30 or 37°C, the agitation speed was controlled at 400 rpm, and the aeration rate was 1 vvm.

### Analysis Procedures

D-sorbitol and l-sorbose in the fermentation broth were evaluated by Shimadzu high-performance liquid chromatography equipped with an Aminex HPX-87H column (Bio-Rad, Hercules, CA, United States) at 40°C with a flow rate of 0.5 ml/min and 5 mmol/L of H_2_SO_4_ as the eluent. A refractive index detector was used for detection (Chen et al., 2019).

## Results

### Effects of D-Sorbitol and Temperature on the Cell Growth of *G. oxydans* WSH-004

**FIGURE 2:**
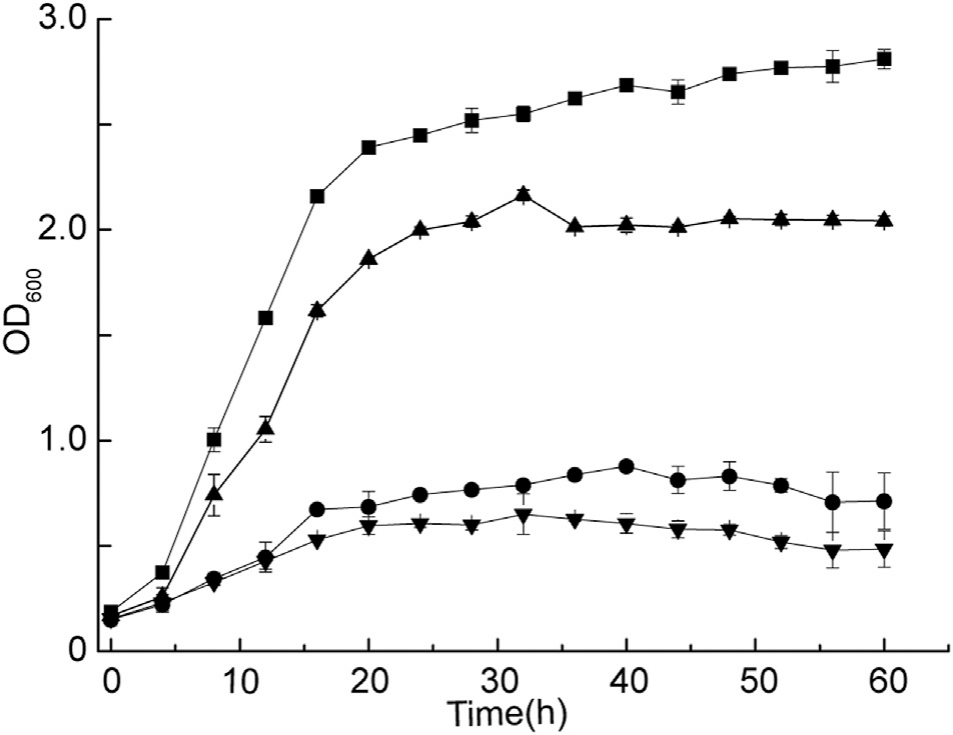
Growth curve of *G. oxydans* with different D-sorbitol concentrations. Square: Growth curve of *G. oxydans* at 30°C in 50 g/L of D-sorbitol medium. Circle: Growth curve of *G. oxydans* at 30°C in 300 g/L of D-sorbitol medium. Up-triangle: Growth curve of *G. oxydans* at 35°C in 50 g/L of D-sorbitol medium. Down-triangle: Growth curve of *G. oxydans* at 35°C in 300 g/L of D-sorbitol medium.

*G. oxydans* WSH-004 was screened from the soil in a previous study (Chen et al., 2019). It directly converted D-sorbitol into 2-KLG without obvious accumulation of by-products during the fermentation process. Therefore, *G. oxydans* WSH-004 had the potential to be a one-step fermentation strain for the production of 2-KLG, which is the direct precursor of vitamin C. *G. oxydans* WSH-004 was grown with different D-sorbitol concentrations (Figure 2). The results showed that the OD_600_ value of *G. oxydans* WSH-004 in the stationary phase could reach 2.7 in 50 g/L of D-sorbitol medium, which was similar to that of most other commonly available *G. oxydans* strains. When the concentration of D-sorbitol reached 300 g/L, which is the commonly used concentration for the industrial-scale production of l-sorbose from D-sorbitol (Zhou et al., 2019), the growth of *G. oxydans* WSH-004 was significantly inhibited with a final OD_600_ value of 0.7. The results showed that the tolerance of *G. oxydans* WSH-004 to high D-sorbitol concentration was poor and should be significantly improved to fulfill the industrial-scale production of l-sorbose and 2-KLG.

### Adaptive Evolution of *G. oxydans* WSH-004 to High D-Sorbitol Concentration

**FIGURE 3:**
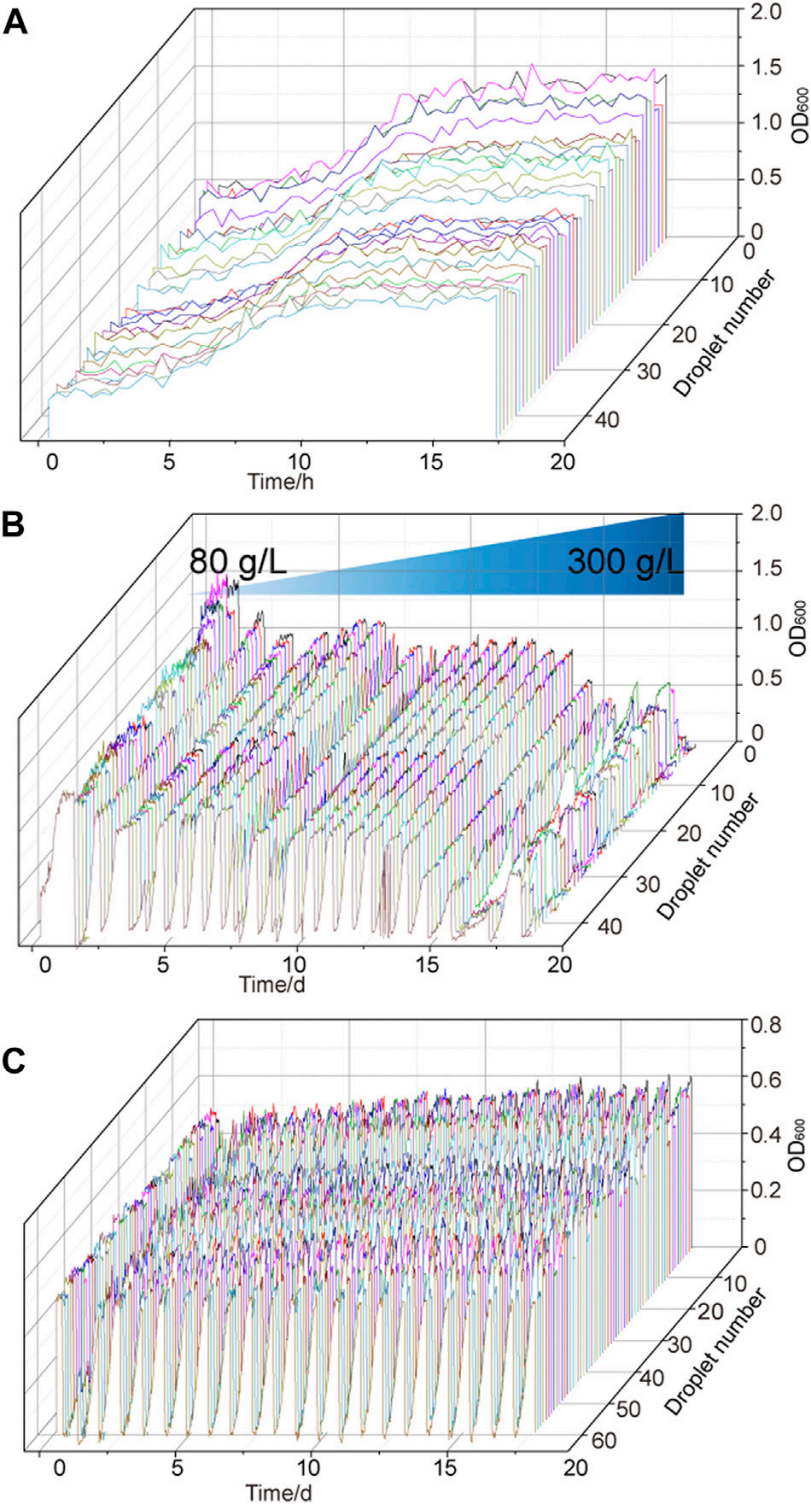
Adaptive evolutionary growth curve of *G. oxydan*s. **(A)** Growth curve of *G. oxydans* WSH-004 in MMC. **(B)** Growth curve of *G.oxydans* in adaptive evolution. **(C)** Adaptive evolution of *G. oxydans* was carried out in a high-concentration D-sorbitol medium. The controlled passage time was 15 h, the culture temperature was 30°C, and the continuous passage of *G. oxydans* in MMC lasted for 15 days.

The strain *G. oxydans* WSH-004 was continuously cultured in the MMC system with different D-sorbitol concentrations to improve the tolerance of the strain to higher D-sorbitol concentrations. The growth curve of *G. oxydans* WSH-004 was first checked in the MMC system. The result showed that the OD_600_ value reached the stationary phase after 10 h with a final OD_600_ value of about 1.2 and a typical growth curve in the MMC system (Figure 3A). The value was different from that in shaking flask fermentation because of the difference in both culture conditions and detection methods. The evolution of tolerance to high D-sorbitol concentration was carried out. The automatic passage time was set to 15 h. After every three passages, the D-sorbitol concentration was automatically increased according to the set program. During the growth of *G. oxydans* WSH-004, the OD_600_ value of every droplet was recorded by MMC (Figure 3B). When the D-sorbitol concentration of *G. oxydans* WSH-004 reached 300 g/L, the OD_600_ values of different drops were quite different. The droplets with a higher OD_600_ value were harvested, cultured in 300 g/L of D-sorbitol medium at 30°C, and then diluted and spread on D-sorbitol plates containing 300 g/L of D-sorbitol. Two single colonies of evolved strains, namely *G. oxydans* MMC1 and MMC2, that could grow well on 300 g/L of D-sorbitol plates, were picked and verified.

*G. oxydans* MMC2 was selected as the starting strain for the second round of adaptive evolution to high D-sorbitol concentration in MMC to stabilize the capacity of *G. oxydans* to tolerate high D-sorbitol concentrations. In the second round of adaptive evolution, no significant growth difference was found among different drops. All drops could be cultured stably in MMC (Figure 3C). After 14 days of continuous culture in MMC, the droplets with higher OD_600_ values were harvested, cultured in 300 g/L of D-sorbitol medium at 30°C, and then diluted and spread on D-sorbitol plates containing 300 g/L of D-sorbitol. Two single colonies of evolved strains, namely *G. oxydans* MMC3 and MMC4, that could grow well on 300 g/L of D-sorbitol plates, were picked and verified.

### Adaptive Evolution of *G. oxydans* to a Higher Temperature

**FIGURE 4:**
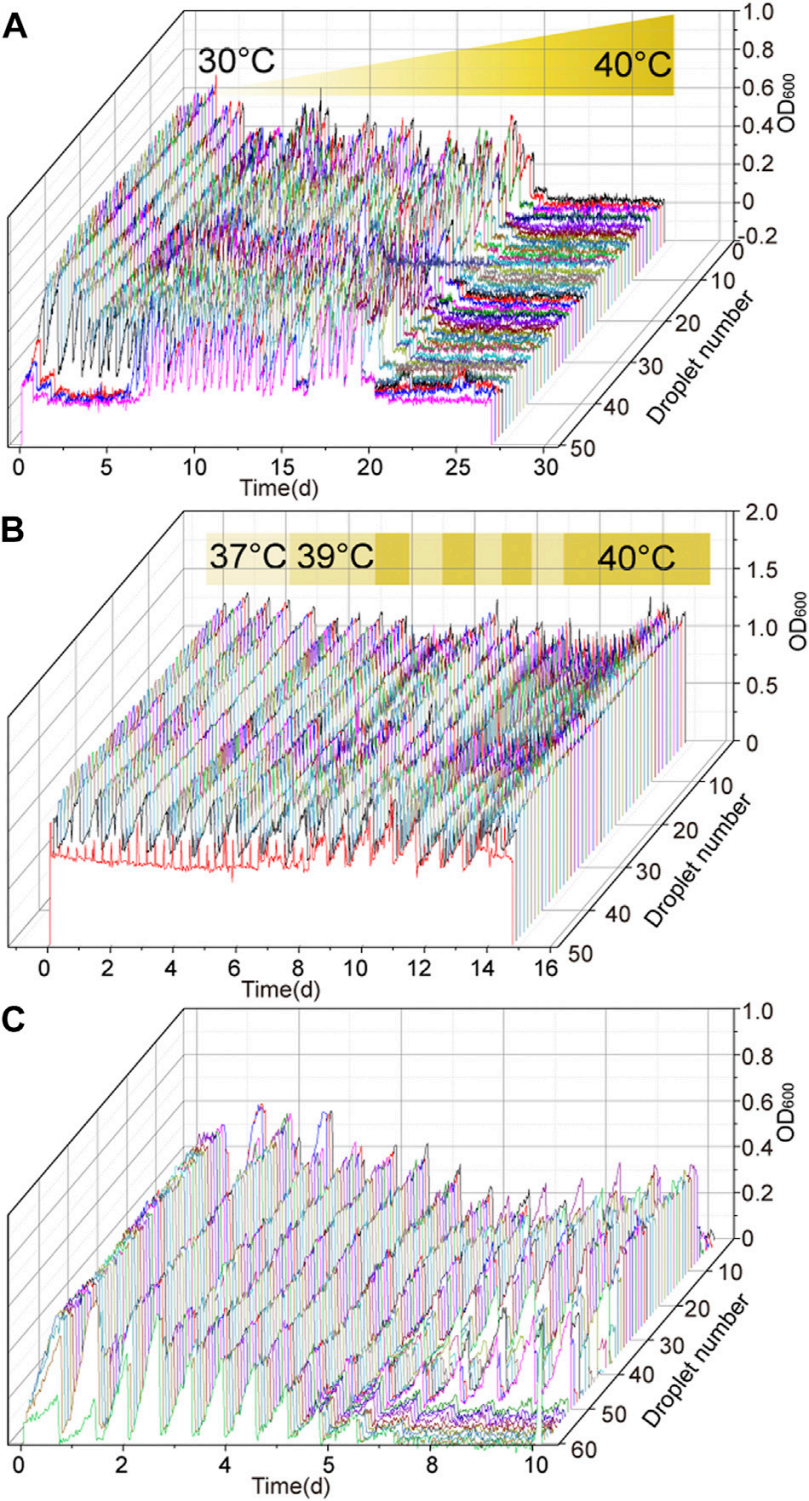
Temperature tolerance adaptive evolution of *G. oxydan*s. **(A)** Adaptability evolution of *G. oxydans* to high temperature: the culture temperature and passage time were changed according to the growth of *G. oxydans* in MMC. **(B)** Adaptability evolution of *G. oxydans* to high temperature: the culture temperature was alternatively switched between 39 and 40°C. **(C)** Adaptability evolution of *G. oxydans* to high temperature: it was continuously cultured at 40°C.

High-temperature fermentation has many advantages in industrial production. In the process of adaptive evolution to high-temperature tolerance, *G. oxydans* MMC4 was used as the starting strain for performing adaptive evolution. The D-sorbitol concentration and culture temperature were initially set at 300 g/L and 30°C, respectively. The result showed that the OD_600_ value could reach to about 0.7 in the MMC system with 300 g/L of D-sorbitol medium at 30°C. Then, the culture temperature was gradually raised from 30 to 40°C (Figure 4), and the D-sorbitol concentration was maintained at 300 g/L. When the incubation temperature was increased to 35°C, the maximum OD_600_ value reached to about 0.5 (Figure 4A). In the later adaptive evolution of temperature tolerance (the temperature was gradually increased from 35 to 39°C), the growth of *G. oxydans* was basically unaffected with a maximum OD_600_ value of about 0.5 (Figure 4A). After 26 days of adaptive evolution in MMC, the droplets with higher OD_600_ value were harvested and then cultured in 300 g/L of D-sorbitol concentration at 39°C. As strains isolated from all drops could not grow at less than 40°C, 39°C was the highest temperature that *G. oxydans* could withstand in this round (Figure 4A). After 27 days of continuous culture in MMC, the droplets with higher OD_600_ value were harvested, cultured in 300 g/L of D-sorbitol medium at 39°C, diluted and spread on D-sorbitol plates containing 300 g/L of D-sorbitol, and cultured at 39°C. Two single colonies of evolved strains, namely *G. oxydans* MMC5 and MMC6, that grew well on 300 g/L of D-sorbitol plates, were picked and verified.

Taking *G. oxydans* MMC6 as the starting strain, the adaptive evolution was further carried out. When the culture temperature increased to 39°C, the evolved strain grew normally (Figure 4B). Then, the evolution temperature was altered between 39 and 40°C (Figure 4B). Finally, when the strains grew stably at 40°C, the droplets with higher OD_600_ value were harvested, cultured in 300 g/L of D-sorbitol medium at 40°C, diluted and spread on D-sorbitol plates containing 300 g/L of D-sorbitol, and cultured at 40°C. Two single colonies of evolved strains, namely *G. oxydans* MMC7 and MMC8, that grew well on 300 g/L of D-sorbitol plates, were picked and verified.

Further adaptive evolution of *G. oxydans* MMC8 was conducted to stabilize the capacity of *G. oxydans* to tolerate both high D-sorbitol concentration and high temperature. The D-sorbitol concentration and culture temperature were initially set at 300 g/L and 37°C, respectively. Once the growth of cells reached the stationary phase, the adaptive evolution temperature was gradually increased from 37 to 40°C (Figure 4C). Finally, when the cells grew stably at 40°C, the droplets with higher OD_600_ value were harvested, cultured in 300 g/L of D-sorbitol medium at 40°C, diluted and spread on D-sorbitol plates containing 300 g/L of D-sorbitol, and cultured at 40°C. Two single colonies of evolved strains, namely *G. oxydans* MMC9 and MMC10, that grew well on 300 g/L of D-sorbitol plates, were picked and verified.

### Verification of Adaptive Evolutionary Strains in Shake Flasks

**FIGURE 5:**
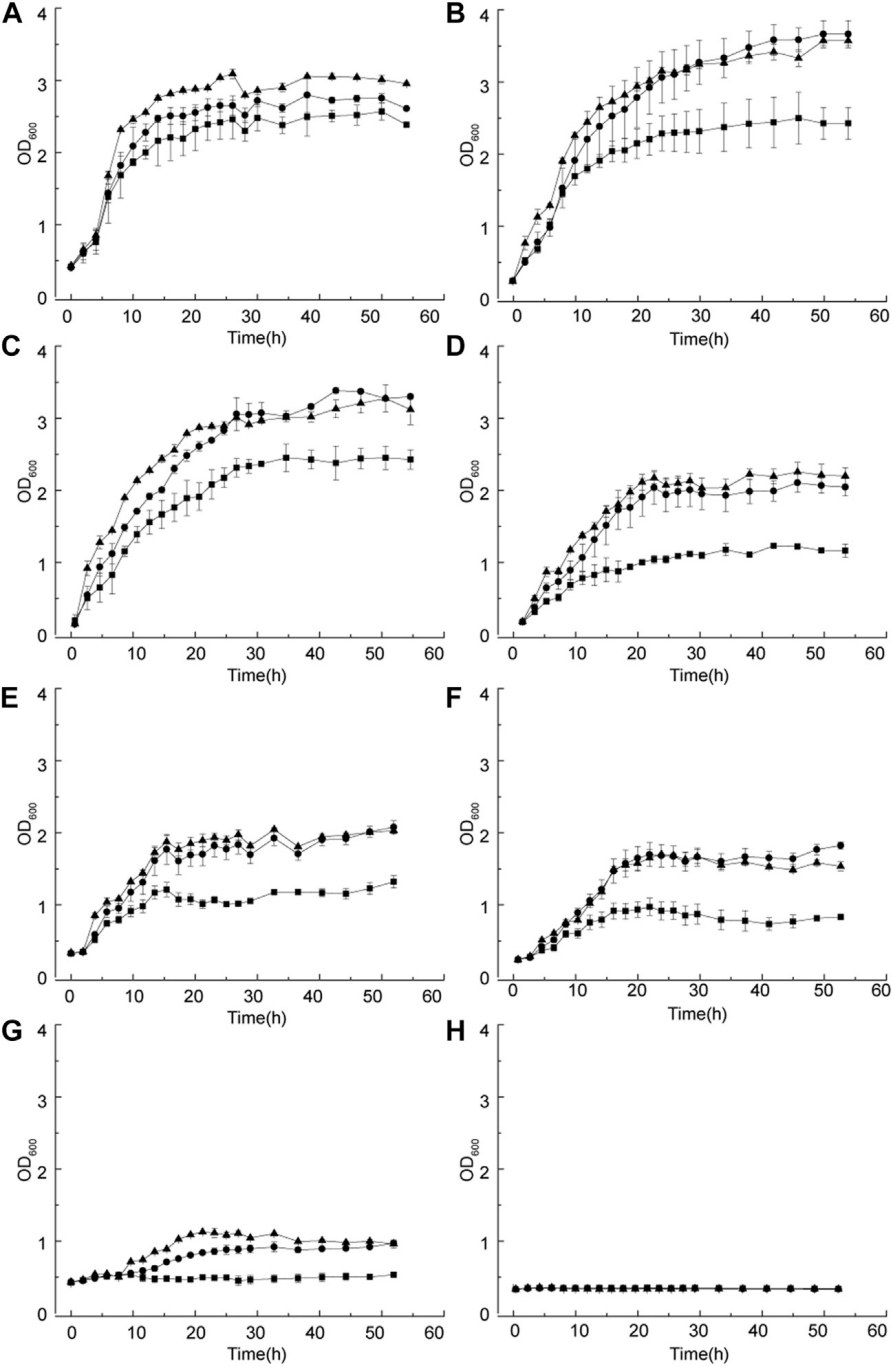
Growth curve of *G. oxydans* with different D-sorbitol concentrations. **(A)** 50 g/L, **(B)** 100 g/L, **(C)** 150 g/L, **(D)** 200 g/L, **(E)** 250 g/L, **(F)** 300 g/L, **(G)** 350 g/L, and **(H)** 400 g/L. Square: *G. oxydans* WSH-004; circle: *G. oxydans* MMC2; and up-triangle: *G. oxydans* MMC4.

Both wild-type and evolved strains were cultured under different D-sorbitol concentrations to verify the D-sorbitol tolerance of the evolved strains. In 50 g/L of D-sorbitol medium, the final OD_600_ values of both *G. oxydans* MMC2 and MMC4 reached to about 2.9, which was higher than that of *G. oxydans* WSH-004 (Figure 5A). *G. oxydans* MMC2 and MMC4 grew better in 100 g/L of D-sorbitol medium than in 50 g/L of the medium. The final OD_600_ values of *G. oxydans* MMC2 and MMC4 was about 3.2 (Figure 4B). In 100–300 g/L of D-sorbitol medium, *G. oxydans* MMC2 and MMC4 grew better than *G. oxydans* WSH-004 (Figures 5C–F). When the D-sorbitol concentration was increased to 350 g/L, *G. oxydans* WSH-004 could not grow, while *G. oxydans* MMC2 and MMC4 grew to a final OD_600_ value of about 1.2 (Figure 5G). When D-sorbitol concentration was increased to 400 g/L, none of the strains could grow (Figure 5H).

**FIGURE 6:**
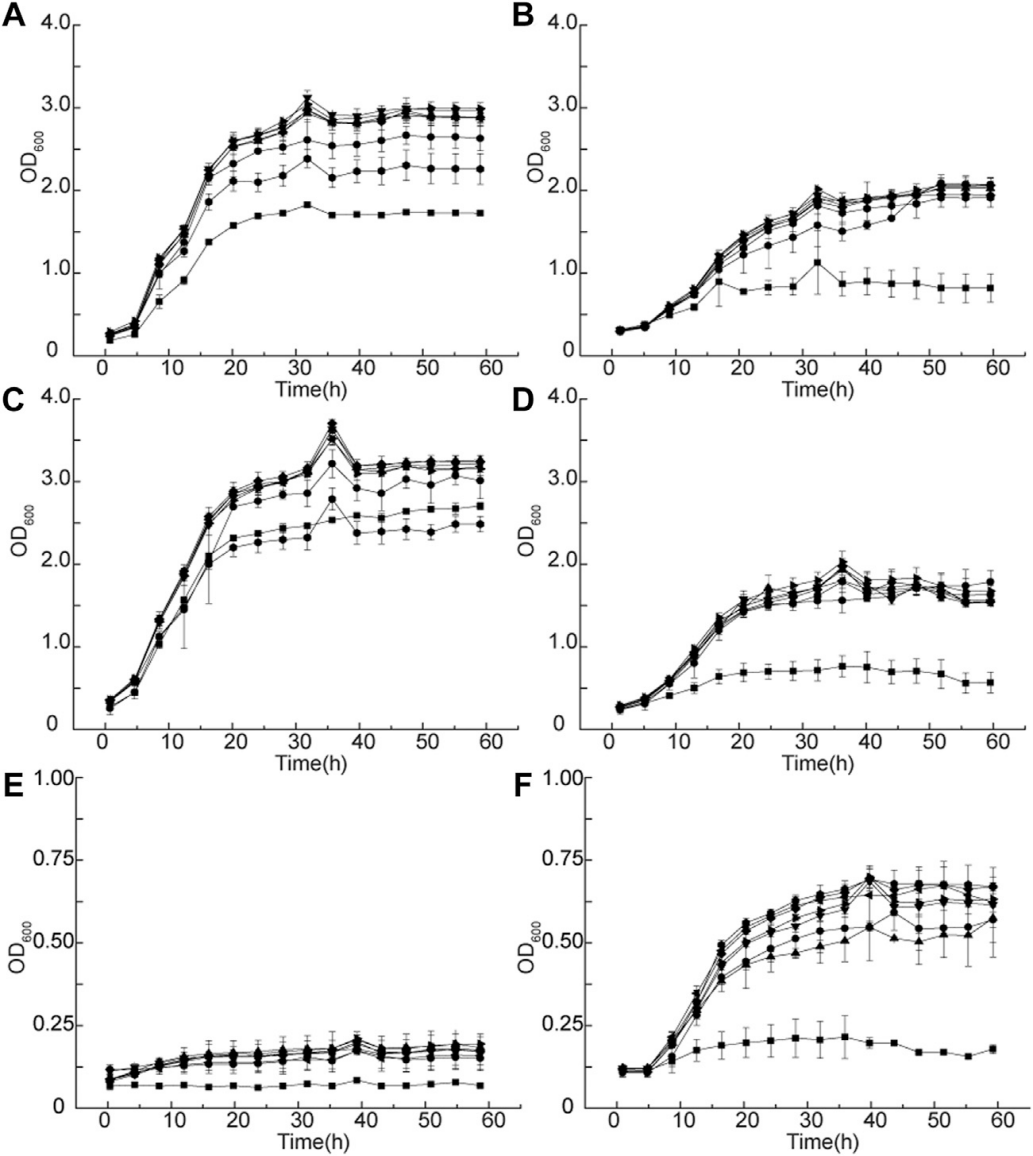
Growth curve of *G. oxydans* at different temperatures. Growth curves at **(A)** 50 g/L of D-sorbitol, 30°C; **(B)** 300 g/L of D-sorbitol, 30°C; **(C)** 50 g/L of D-sorbitol, 35°C; **(D)** 300 g/L of D-sorbitol, 35°C; **(E)** 50 g/L of D-sorbitol, 40°C; and **(F)** 300 g/L of D-sorbitol, 40°C. Square: *G. oxydans* WSH-004; circle: *G. oxydans* MMC4; up-triangle: *G. oxydans* MMC5; down-triangle: *G. oxydans* MMC6; rhombus: *G. oxydans* MMC7; left triangle: *G. oxydans* MMC8; right triangle: *G. oxydans* MMC9; and pentagon: *G. oxydans* MMC10.

*G. oxydans* MMC4-MMC10 grew better than the wild-type strain in the 50 g/L D-sorbitol media at 30 and 35°C, and the OD_600_ value of the evolved strains (about 3.5) was higher than that of the wild-type strains (about 2.0) (Figures 6A-D). When the temperature was 40°C, the growth of all strains was inhibited in 50 g/L of D-sorbitol medium (Figure 6E). However, 300 g/L of D-sorbitol could benefit cell growth at 40°C. The evolved strains grew much better at 40°C than in 50 g/L of D-sorbitol medium. The final OD_600_ value reached to about 0.75 (Figure 6F). Therefore, high D-sorbitol concentration could protect *G. oxydans* under high-temperature conditions.

### Bioreactor Fermentation for l-Sorbose Synthesis

**FIGURE 7:**
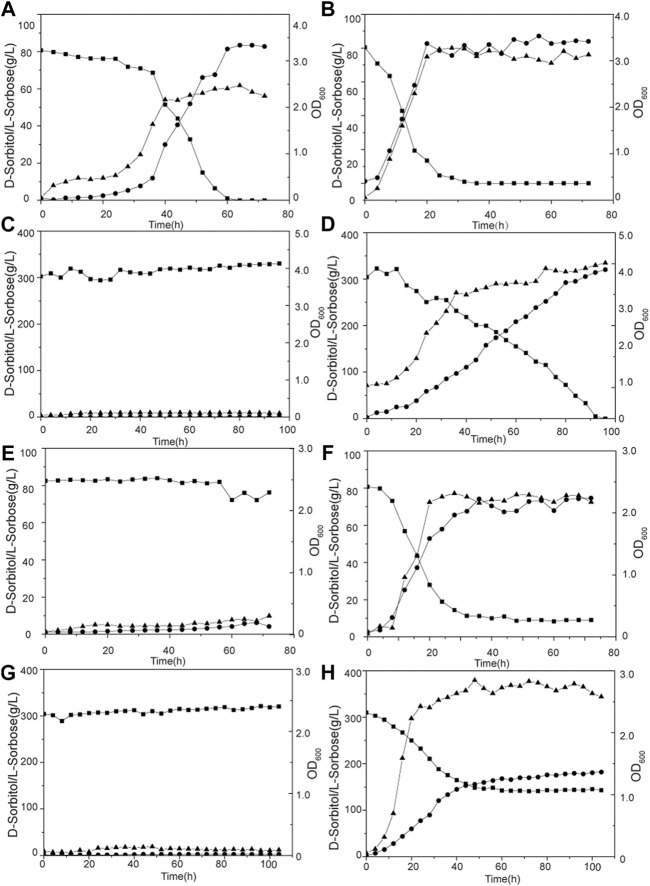
Production of l-sorbose by *G. oxydans* in a 5-L fermentor. **(A)** *G. oxydans* WSH-004 in 80 g/L of D-sorbitol at 30°C; **(B)** *G. oxydans* MMC10 in 80 g/L of D-sorbitol at 30°C; **(C)** *G. oxydans* WSH-004 in 300 g/L of D-sorbitol at 30°C; **(D)** *G. oxydans* MMC10 in 300 g/L of D-sorbitol at 30°C; **(E)** *G. oxydans* WSH-004 in 80 g/L of D-sorbitol at 37°C; **(F)** *G. oxydans* MMC10 in 80 g/L of D-sorbitol at 37°C; **(G)** *G. oxydans* WSH-004 in 300 g/L of D-sorbitol at 37°C; and **(H)** *G. oxydans* MMC10 in 300 g/L of D-sorbitol at 37°C. Square: D-sorbitol; circle: l-sorbose; and up-triangle: OD_600_.

Batch fermentation of *G. oxydans* WSH-004 and evolved strain *G. oxydans* MMC10 was conducted in a 5-L bioreactor to verify the production capacity of l-sorbose. Both *G. oxydans* WSH-004 and *G. oxydans* MMC10 could completely convert 80 g/L of D-sorbitol into l-sorbose at 30°C. However, the required fermentation time was quite different. It took about 60 and 25 h for *G. oxydans* WSH-004 and *G. oxydans* MMC10, respectively, to completely convert the D-sorbitol to l-sorbose, with productivities of 1.33 g/(Lh) and 3.20 g/(Lh), respectively (Figures 7A,B). When the concentration of D-sorbitol was increased to 300 g/L, the growth of *G. oxydans* WSH-004 was completely inhibited, while *G. oxydans* MMC10 could still completely convert D-sorbitol into l-sorbose in 96 h (Figures 7C,D). The productivity of *G. oxydans* MMC10 with the 300 g/L of D-sorbitol fermentation medium was 3.13 g/(Lh), which was similar to that in the 80 g/L of D-sorbitol fermentation medium.

Then, the fermentation temperature was raised to 37°C. *G. oxydans* MMC10 grew normally, while *G. oxydans* WSH-004 could not grow with 80 g/L or 300 g/L of D-sorbitol (Figures 7E,F). *G. oxydans* MMC10 could not completely convert D-sorbitol into l-sorbose at 37°C in 80 g/L of D-sorbitol medium. The final concentration of l-sorbose was 74 g/L at 35 h, with a productivity of 2.11 g/(Lh). Besides, *G. oxydans* MMC10 could not completely convert D-sorbitol into l-sorbose in 300 g/L of D-sorbitol medium at 37°C. However, the strain grew better than in the 80 g/L of D-sorbitol medium, and the OD_600_ value reached to 2.8. The final l-sorbose concentration was 160 g/L after 60 h, with a productivity of 2.67 g/(Lh) (Figures 7G,H). It was presumed that the enzyme activity and stability of D-sorbitol dehydrogenase were affected under high-temperature conditions.

### Comparative Genomic Analysis of *G. oxydans*

**FIGURE 8:**
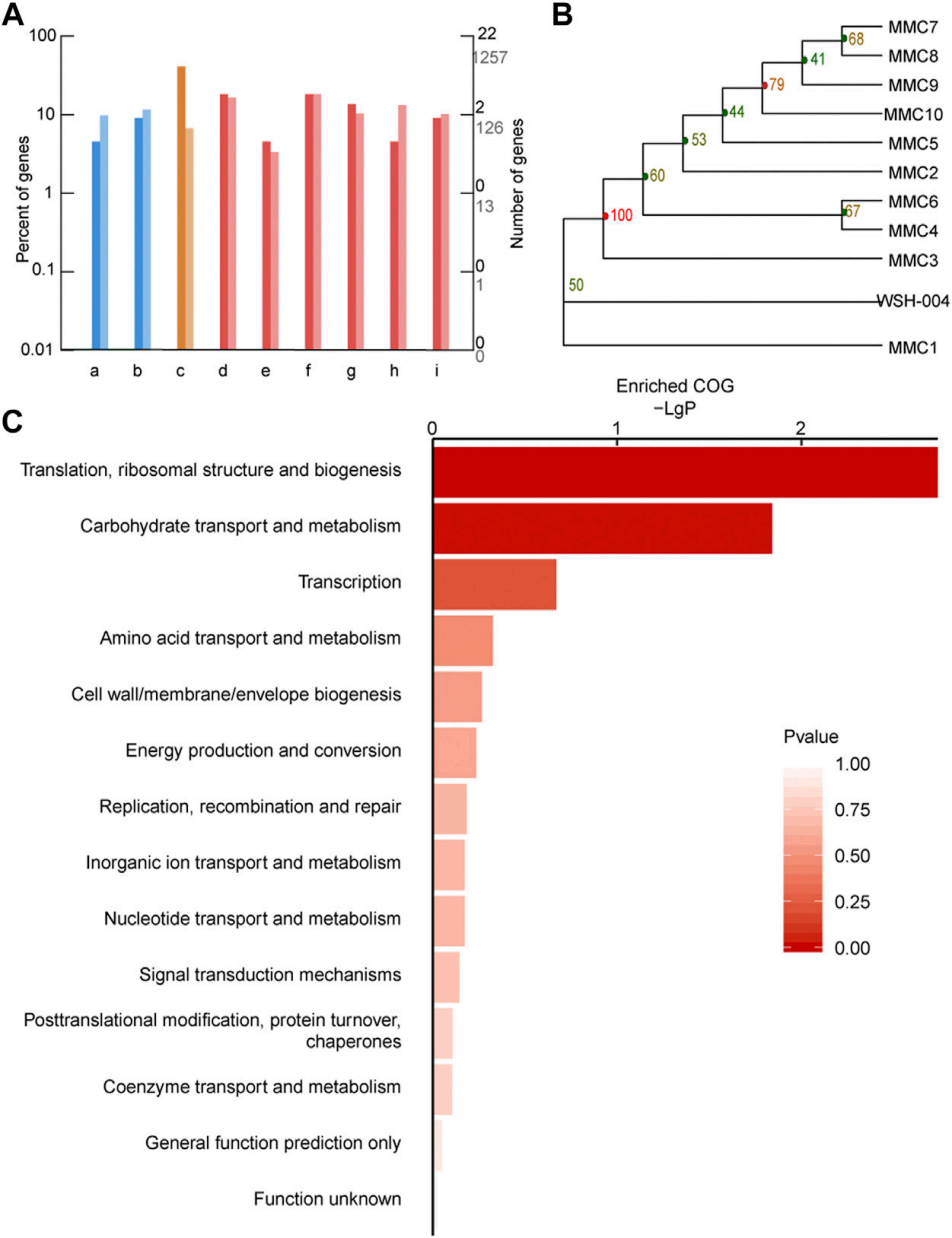
Comparative genomic analysis of *G. oxydans* strains. **(A)** GO annotation classification column map of variant genes. The horizontal axis is the functional classification, and the vertical axis is the number of genes in the classification **(right)** and the percentage of the total number of genes on the annotation **(left)**. Different colors represent different categories. The dark color on the histogram and the axis represents the variant gene, and the light color represents all genes (background genes). (i) Membrane transport, (ii) signal transduction, (iii) translation, (iv) amino acid metabolism, (v) biosynthesis of other secondary metabolites, (vi) carbohydrate metabolism, (vii) energy metabolism, (viii) metabolism of cofactors and vitamins, and (i) nucleotide metabolism. **(B)** Phylogenetic tree of a series of adaptive evolution strains. *G. oxydans* phylogenetic tree based on the SNP of each sample. **(C)** Significant enrichment function bar chart. The vertical axis represents function annotation information, and the horizontal axis represents the degree of enrichment corresponding to the function, that is, the −log10 (*p* value) value. The higher the enrichment degree, the longer the column and the darker the color.

Genome sequencing was performed on evolved strains to identify mutations that might contribute to the improved phenotypes in the evolved populations. Based on the comparative genomic analysis, 104 mutations were detected among the ten evolved strains comprising 53 point mutations and 51 insertions or deletions. The genetic variations have been listed in Supplementary Material 1 and Supplementary Material 2. Functional annotations of the mutated genes (Figure 8A) showed that the mutated genes were mainly related to translation (ribosomal small subunits S17 and S3, and the major ribosomal subunits L2, L3, L6, L24, and L29), amino acid and nucleotide metabolism, carbohydrate metabolism, and energy metabolism. In the same time, all evolved strains have the ribosome mutation-related genes and this gene was listed in Table 1. A phylogenetic tree of evolved strains and wild-type strains was built based on the SNP of each sample using the adjacency NJ algorithm (Figure 8B). According to the clusterProfiler for feature enrichment analysis, mutant genes were mainly involved in ribosomes, ribosomal protein complexes, and protein mutations that constituted ribosomes (Figure 8C).

**TABLE 1:** The ribosome mutation-related genes of evolved strains.

Description	Position	Reference	Changed	Mutation type
50S-L6	396,338	ACG	A	frameshift_variant
50S-L6	396,346	C	CT	frameshift_variant
50S-L6	396,849	T	TG	frameshift_variant
50S-L6	396,885	C	CG	frameshift_variant
50S-L24	398,286	C	CT	frameshift_variant
30S-S17	399,027	C	CGT	frameshift_variant
30S-S17	399,027	C	CGT	frameshift_variant
50S-L29	399,621	A	AAG	frameshift_variant
30S-S3	400,096	CG	C	frameshift_variant
50S-L2	401,397	CT	C	frameshift_variant
50S-L2	401,419	A	AC	frameshift_variant
50S-L23	402,284	A	T	missense_variant
30S-S10	404,002	G	T	missense_variant

## Discussion

In a previous study, a strain *G. oxydans* WSH-004 could directly produce 2-KLG from D-sorbitol by a high-throughput screening method based on 2-keto-gulonic acid dehydrogenase (Chen et al., 2019). In the present study, adaptive evolution of *G. oxydans* WSH-004 was conducted using an automated, high-throughput microbial cultivation MMC system (Jian et al., 2020) to improve its tolerance to high temperature and high D-sorbitol concentration. A series of evolved strains were obtained within 90 days. These strains achieved significantly enhanced growth and conversion rates and productivity with higher substrate concentration, and at higher temperature compared with *G. oxydans* WSH-004. Analysis of the genome of *G. oxydans* WSH-004 and the evolved strains showed that mutated genes were mainly related to translation, thus providing new research directions for the tolerance mechanism of *G. oxydans*. The obtained results suggested that the MMC system greatly improved the efficiency of the adaptive evolution of microorganisms and obviously reduced labor and cost involved in the adaptive evolution process.

The substrate/product resistance of microorganisms is vital for their applications in industrial fermentation processes (Mohamed et al., 2020; Wang et al., 2021). In recent years, many reports on substrate/product tolerance of microbial strains, such as *Clostridium acetobutylicum* and *Escherichia coli* (Alsaker et al., 2010; Zhang et al., 2015), have been published, but studies on the tolerance of *G. oxydans* are few. Mechanisms of substrate/product stress have been studied mostly in Gram-negative microorganisms. For example, Si et al. (2016) found a series of key genes related to organic solvent tolerance of *Escherichia coli* through gene chip technology, and Zhang et al. (2015) constructed a global transcription factor mutation library and finally screened two strains with high organic solvent tolerance. In the present study, D-sorbitol tolerant strains were obtained through adaptive evolution in MMC, and the genomes of D-sorbitol-tolerant strains were re-sequenced. The findings showed that the mutated genes were mainly related to translation. However, due to the difficulty in genetic modification, future studies need to verify these mutation sites for enhancing specific phenotypes rather than simply modifying the membrane components or other known processes.

High-temperature fermentation has great advantages in industrial production, especially for reducing cooling costs and risk of contaminations, and simultaneous saccharification and fermentation of pretreated lignocellulosic for biofuel production (Zhang et al., 2016; Vishnu Prasad et al., 2020). However, most of the strains produced in the industry have poor heat resistance. Fermentation process at 30°C could be a big challenge for cooling water supply in a majority of temperate, subtropical, and tropical regions. Previous studies have demonstrated the mechanism of heat resistance in bacteria, such as reduction of the level of intracellular reactive oxygen species (Xu et al., 2018; Qin et al., 2020), regulation of chaperones for maintaining protein-folding homeostasis (Kim et al., 2020), and mutation of F-type ATPase (Huang et al., 2018). Some of the thermotolerant *Gluconobacter* strains, which can withstand a temperature of 36–37°C, have been isolated for producing 5-keto-d-gluconic acid or coenzyme Q_10_ (Saichana et al., 2009; Moghadami et al., 2019). However, the specific heat resistance mechanism remains unclear. In this study, *G. oxydans* MMC10 could withstand 40°C in 300 g/L of D-sorbitol medium through adaptive evolution in MMC. The identified mutations related to thermotolerance could be useful for further rational engineering of thermotolerant bacteria to facilitate their applications in industrial fermentation processes.

Many strategies can be applied to improve the specific phenotypes of microorganisms for industrial applications, such as metabolic engineering (Tian et al., 2020), mutagenesis screening (Jiang et al., 2018), and adaptive evolution (Cubas-Cano et al., 2019), with their own merits and demerits. Compared with metabolic engineering, adaptive evolution is a simple and efficient way to improve microbial tolerance because identifying gene targets for tolerance remains an overwhelming challenge and microbial metabolic networks complexity and interconnectivity. However, conventional adaptive evolution for microorganisms is typically time-consuming and labor-intensive, for example, 420 days for improving the conversion rate of nonglucose sugars of *G. oxydans* (Jin et al., 2019), 150 days for improving the salinity resistance of *Schizochytrium sp*. (Sun et al., 2018), 200 days for co-utilization of glucose and xylose of *Zymomonas mobilis* (Sarkar et al., 2020), and 230 generations for enhanced native methanol assimilation in *S. cerevisiae* (Espinosa et al., 2020). Reports on enhanced heat resistance by adaptive evolution or other rational strategies are rare (Cox et al., 2010). In this study, tolerant *G. oxydans* strains could be obtained within 90 days because the MMC system provided an automated, high-throughput microbial cultivation approach for adaptive evolution. Meanwhile, the growth conditions could be easily evaluated by an online detection system. Besides, the automated process could greatly facilitate the performance of experimental capabilities, throughput, clarity, and repeatability.

In conclusion, *G. oxydans* strains with enhanced tolerance to higher D-sorbitol concentration and higher temperature were obtained in only 90 days using an established continuous MMC system. Compared with the wild-type strain, *G. oxydans* WSH-004 not only developed tolerance to higher substrate concentration and higher temperature but also showed significantly improved titer, yield, and productivity under different conditions. The results proved that the droplet-based adaptive evolution system was a powerful tool to obtain the target phenotypes in acceptable durations. Comparative genomic analysis of evolved strains showed that the mutations were mainly located on ribosomal subunits. Further systematic investigation should be performed to reveal the mechanism underlying the phenotypes because of the complexity in establishing the relationships between the genotype and the phenotype at a global translational level.

## Data Availability

The original contributions presented in the study are included in the article/Supplementary Material, further inquiries can be directed to the corresponding author.
